# Targeted Expression of Retinoschisin by Retinal Bipolar Cells in XLRS Promotes Resolution of Retinoschisis Cysts Sans RS1 From Photoreceptors

**DOI:** 10.1167/iovs.63.11.8

**Published:** 2022-10-13

**Authors:** Camasamudram Vijayasarathy, Yong Zeng, Dario Marangoni, Lijin Dong, Zhuo-Hua Pan, Elizabeth M. Simpson, Robert N. Fariss, Paul A. Sieving

**Affiliations:** 1 Section for Translational Research in Retinal and Macular Degeneration, National Institutes of Health, Bethesda, Maryland, United States; 2 Genetic Engineering Facility, National Eye Institute, National Institutes of Health, Bethesda, Maryland, United States; 3 Biological Imaging Core, National Eye Institute, National Institutes of Health, Bethesda, Maryland, United States; 4 Department of Ophthalmology, Visual and Anatomical Sciences, Wayne State University School of Medicine, Detroit, Michigan, United States; 5 Centre for Molecular Medicine and Therapeutics at BC Children's Hospital, University of British Columbia, Vancouver, British Columbia, Canada; 6 Center for Ocular Regenerative Therapy, Department of Ophthalmology, University of California Davis, United States

**Keywords:** X-linked retinoschisis, schisis, bipolar cells, cell adhesion, photoreceptors

## Abstract

**Purpose:**

Loss of retinoschisin (RS1) function underlies X-linked retinoschisis (XLRS) pathology. In the retina, both photoreceptor inner segments and bipolar cells express RS1. However, the loss of RS1 function causes schisis primarily in the inner retina. To understand these cell type–specific phenotypes, we decoupled RS1 effects in bipolar cells from that in photoreceptors.

**Methods:**

Bipolar cell transgene RS1 expression was achieved using two inner retina–specific promoters: (1) a minimal promoter engineered from glutamate receptor, metabotropic glutamate receptor 6 gene (mini-mGluR6/ Grm6) and (2) MiniPromoter (Ple155). Adeno-associated virus vectors encoding RS1 gene under either the mini-mGluR6 or Ple-155 promoter were delivered to the XLRS mouse retina through intravitreal or subretinal injection on postnatal day 14. Retinal structure and function were assessed 5 weeks later: immunohistochemistry for morphological characterization, optical coherence tomography and electroretinography (ERG) for structural and functional evaluation.

**Results:**

Immunohistochemical analysis of RS1expression showed that expression with the MiniPromoter (Ple155) was heavily enriched in bipolar cells. Despite variations in vector penetrance and gene transfer efficiency across the injected retinas, those retinal areas with robust bipolar cell RS1 expression showed tightly packed bipolar cells with fewer cavities and marked improvement in inner retinal structure and synaptic function as judged by optical coherence tomography and electroretinography, respectively.

**Conclusions:**

These results demonstrate that RS1 gene expression primarily in bipolar cells of the XLRS mouse retina, independent of photoreceptor expression, can ameliorate retinoschisis structural pathology and provide further evidence of RS1 role in cell adhesion.

X-linked retinoschisis (XLRS) is an early onset, bilateral, X-linked recessive retinal and macular degeneration seen in young boys and men with an estimated prevalence of 1:5000 to 1:25,000. XLRS results from loss-of-function mutations in the retinoschisis (***RS1***) gene.^1^^,^^2^ It is characterized by early-onset impairment of central vision from bilateral foveomacular cystic cavities that involve the inner retina^1^ and additional retinal layers. Many affected males also exhibit peripheral retinoschisis, which increases the risk of retinal detachment (Fig. 1A). Schisis involving the inner retinal layers disrupts visual signal transmission from photoreceptor cells to optic nerve head bipolar cells and leads to slow but progressive decline in vision with age. Electrical response of the dark-adapted retina on the electroretinogram (ERG), show characteristic decreases in the b-wave amplitude compared with a relative preservation of the initial negative a-wave, termed the so-called electronegative ERG, indicating inner retinal abnormalities (Fig. 1B). There is considerable heterogeneity between mild and more severe phenotypes, and the rates of progression can differ markedly and lack correlation with genotype. Currently, there is no medical treatment for XLRS. Carbonic anhydrase inhibitors have been tried in patients with XLRS for macular cyst-like lesions, but with modest and variable results. Recombinant adeno-associated virus (rAAV)–mediated RS1 gene replacement therapy is being explored (NIH: ClinicalTrials.gov: NCT02317887 and AGTC Inc. ClinicalTrials.gov: NCT02416622).^3^^–^^5^ The clinical pathology, molecular and cell biology of XLRS and the strategies to treat XLRS have been reviewed comprehensively elsewhere.^6^^–^^11^

**Figure 1.**
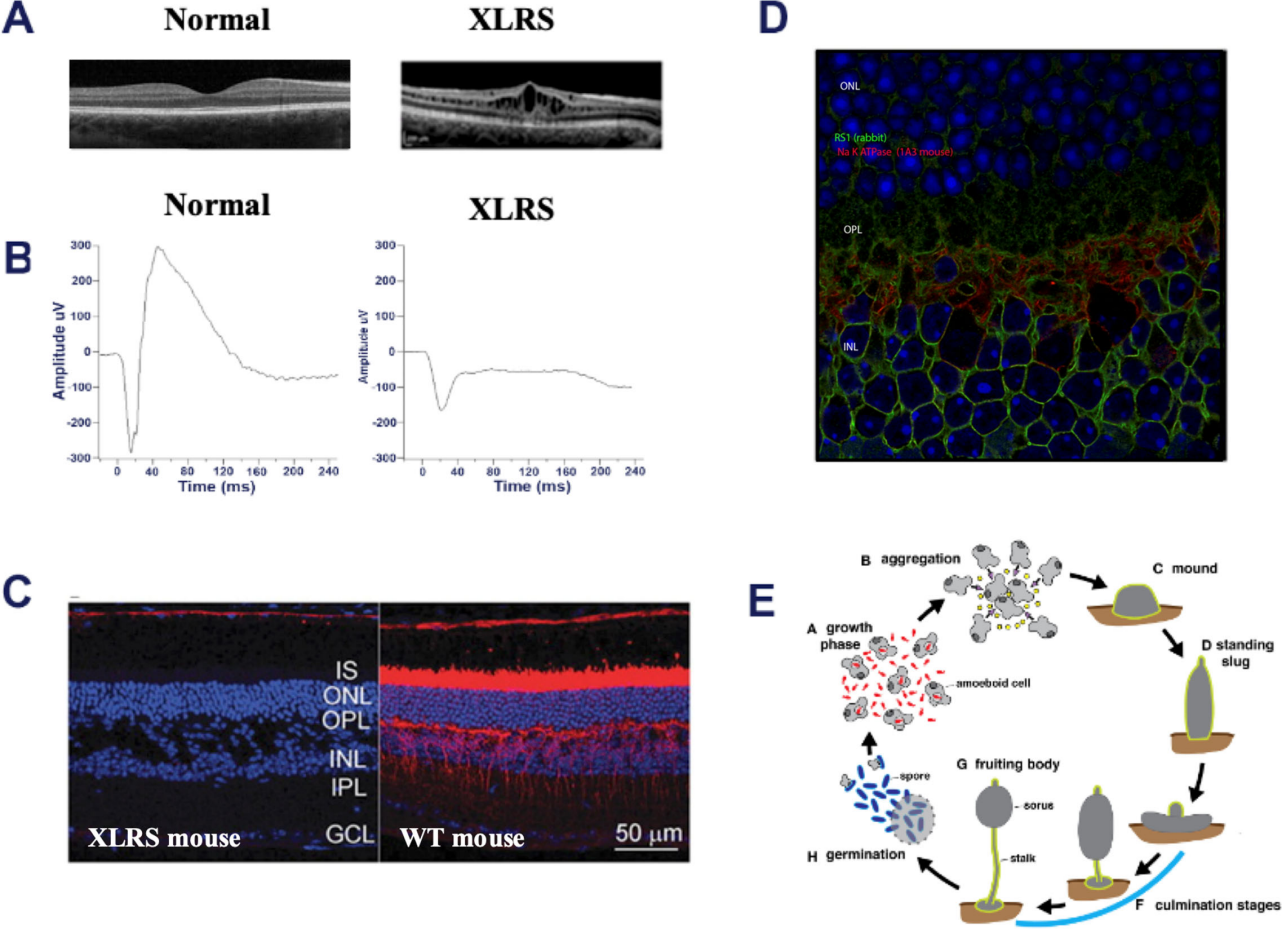
**Human and mouse XLRS**. (**A**) Retina photograph of a 17-year-old XLRS subject with 20/63 acuity showing classic macular retinoschisis with a subtle spoke wheel pattern radiating from the fovea. (**B**) Representative dark-adapted ERG combined responses (arising from photoreceptors and bipolar cells) of the XLRS subject show characteristic b-wave reduction disproportionate to a-wave reduction. (**C**) RS1 expression in mouse retina and XLRS phenotype. Retinal cryosections were immunolabeled with antibodies against RS1 (*red*, 1:1000); Na/K ATPase a3 subunit (*green*, 1:1000). The nuclei were counterstained with DAPI (*blue*). In WT retina RS1 is profusely expressed in photoreceptor IS and in inner retinal layers. In XLRS mouse retina, loss of Rs1 expression results in splitting of the inner retina cell layers. (**D**) RS1 expression on mouse retina bipolar cells and its colocalization with Na/K-ATPase, a plasma membrane marker. (E) Discoidin domain has been shown to be involved in cell adhesion during the streaming and aggregation of *D.* *discoideum*. During the growth phase of development, *D.* *discoideum* amoeboid cells feed on bacteria and replicate by binary fission. The development cycle is initiated upon starvation (resource depletion), and aggregation occurs when starving cells secrete cyclic AMP to recruit additional cells. Discoidin I is synthesized profusely as cells stream together into aggregate to form slug and fruiting body. OPL, outer plexiform layer; IPL, inner plexiform layer; GCL, ganglion cell layer. Fig 1E is reproduced from Dunn JD, Bosmani C, Barisch C, et al. Eat prey, live: *Dictyostelium discoideum* as a model for cell-autonomous defenses. *Front Immunol*. 2018;8:1906. Copyright © 2018 Dunn, Bosmani, Barisch, Raykov, Lefrançois, Cardenal-Muñoz, López-Jiménez and Soldati; open-access, distributed under the terms of the Creative Commons Attribution License (CC BY).

Genetically engineered XLRS rodent models (*Rs1* gene knockout) recapitulate aspects of schisis pathology observed in the human disease and are critical for understanding disease pathogenesis.^6^ These models supported preclinical studies for rAAV8 *RS1* gene transfer into XLRS mouse retina (Fig. 1C). Retinoschisin (RS1) is a cell surface protein expressed primarily in the retina and the pineal gland.^12^^–^^14^ In the adult retina, RS1 is synthesized in rods, cones, and bipolar cells (Figs. 1C and D), and after synthesis it is secreted into the extracellular space, where it remains bound to the external leaflet of the plasma membrane and in the synaptic regions.^15^ The 24kDa RS1 protein encodes 157 amino acids conserved discoidin domain sequence, named for its homology to lectin discoidin I, a protein secreted by the slime mold *Dictyostelium discoideum* (Fig. 1E). Discoidin domain is involved in cell–cell adhesion,^16^ and the schisis pathology observed in XLRS has been attributed to the loss of RS1 function as a retinal cell–cell adhesion protein.^1^^,^^17^ Paired octamer rings of RS1 structure suggest a junctional model for cell–cell adhesion, but there is no direct in vivo evidence that RS1 is a junctional adhesion molecule.^18^^–^^20^ However, the XLRS schisis pathology (splitting of inner retinal layers) suggests adhesion as a fact by implication or inference. Most important, the replacement of a functional RS1 gene into XLRS mouse retina via AAV-mediated RS1 gene therapy results in closely packed assemblage of retinal cells, the disappearance of schisis cavities, and the restoration of synaptic function, implying adhesion as a possible mechanism.^21^

The most common finding in XLRS is extensive retinal splitting (schisis formation), primarily in the inner nuclear layer (INL) in 90% of patients with XLRS and the outer nuclear and plexiform layers in a few others.^11^^,^^22^^,^^23^ In XLRS mouse models, the photoreceptors localized in the outer nuclear layer (ONL) undergo extensive cell death and this was attributed to the loss of cell adhesion in the INLs.^17^^,^^24^ It is not clear whether the production of cell type–specific phenotypes from the same genotype is a developmental variation or local microenvironmental effects. To understand cell-specific phenotype differences, and to probe the causes and consequences, we explored tools to decouple RS1 function in the bipolar cells from effects in photoreceptors. AAV-mediated transduction and cell-specific transgene expression in bipolar cells was achieved using two inner retina–specific promoters: (1) a minimal promoter engineered from glutamate receptor, metabotropic glutamate receptor 6 gene (mini-mGluR6/Grm6),^25^ and (2) an rAAV-compatible MiniPromoter (Ple155) that gives restricted bipolar cell expression in the retina.^26^^–^^29^ We anticipated that targeting RS1 expression in XLRS mouse bipolar neurons under these promoters would help to clarify whether this bipolar selective expression would prevent schisis-related pathology of the inner retina independent of photoreceptor function.

## Methods

This study was performed according to protocols approved by the National Institute of Health IACUC standards.

### Animals

Age-matched C57BL/6 wild-type (WT) and XLRS (RS1 gene knockout: Rs1-KO) mice aged 7 days to 12 months were used in the study. XLRS mice were generated in our laboratory at the National Institutes of Health (NIH) from C57BL/6 blastocytes and backcrossed onto the C57BL/6J strain (The Jackson Laboratory, Bar Harbor, ME, USA) as previously described in detail. The phenotype and natural history of this animal have been carefully described.^30^^–^^33^ Animal experiments were conducted in accordance with the National Institutes of Health Animal Care and Use Committee, the ARVO Statement for the Use of Animals in Ophthalmic, and Vision Research and NIH Institutional Biosafety Committee protocols (NIH/National Eye Institute Protocol Number NEI-617; Human Pathogen Registration Document [HPRD] #4766; Recombinant DNA Registration Document RD-09-II-03).

### Bipolar Cell–Specific Promoters

To target RS1 gene expression specific to bipolar cells, we vetted two rAAV gene transfer vectors with bipolar cell–specific promoters: Mini-mGluR6: rAAV2/2 Y444F ITR-In4s-In3-200En-mGluR500P-RS1-hGHpA-ITR and rAAV8 ITR-Ple155-Intron-RS1-WPRE-ITR, where rAAV2/2-rAAV is a type 2/serotype 2; rAAV8 is recombinant adeno‑associated virus 8; ITR is the inverted terminal repeat; WRPE is Woodchuck hepatitis virus posttranscriptional regulatory element; and hGHpA is human growth hormone poly adenylation sequence (Supplementary Figs. S1, S2).

#### Improved Mini-mGluR6 Promoter

This promoter is constructed and modified in part from the well-known, ON bipolar cell 9.5 kb upstream sequence of mGluR6 promoter.^34^ The 9.5-kb sequence is too long for packaging into rAAV vectors (maximum capacity of approximately 4.7 kb), and shorter versions were developed through bioinformatic analyses. The specificity of the promoter was tested by rAAV-mediated delivery via intravitreal administration into the mouse or marmoset monkey retina for targeted transgene expression in bipolar neurons.^25^ This 2.2-kb optimal promoter construct, In4s-In3-200En-mGluR500P (mini-mGluR6), contained the 200-bp enhancer,^35^ a shortened 500-bp mGluR6 promoter, and two intronic sequences. The fluorescent reporter construct with these sequences, that is, rAAV2/2-Y444-In4sIn3-200En-mGluR500P-mCherry, has been reported previously (Supplementary Fig. S1).^25^ We cloned RS1 cDNA downstream of this improved mini-promoter (In4s-In3-200En-mGluR500P, from here on referred to as mini-mGluR6 promoter by replacing mCherry with RS1 at SacI and XhoI restriction sites). Viral vectors were made by packaging the plasmid construct into rAAV2 serotype 2 with a single capsid mutation-Y444F (Virovek, Hayward, CA, USA).

#### MiniPromoter Ple155

Simpson et al. have developed numerous rAAV optimized cell type restricted human-DNA MiniPromoters for expression in the brain and eye.^26^^–^^29^^,^^36^^,^^37^ These MiniPromoters were developed by computational analysis of human and mouse genomic DNA sequences and tested for target cell specific expression in vivo. For expression in ON bipolar cells of the mouse retina, this work focused on testing alternative promoters and regulatory regions of the Purkinje cell protein 2 (PCP2) gene, well-known for endogenous ON bipolar cell expression.^38^^–^^40^ The resulting MiniPromoter used here was Ple155, which drove expression from rAAV that was restricted to ON bipolar cells and was shown to be independent of serotype and delivery method.^27^^,^^28^ Ple155 virus genome plasmid pEMS2229 carrying ssAAV-Ple155-EmGFP-WPRE (ss, single stranded; EmGFP emerald green fluorescent protein; WPRE) was provided by Simpson. For cloning Ple155-RS1, we replaced EmGFP with human RS1 cDNA sequence at the Nco1/Bsp1407I restriction sites of the viral vector plasmid DNA (Supplementary Fig. S2). The virus vector was made by packaging into AAV serotype 8, from now on referred to as rAAV8-Ple155-RS1 [Virovek]).

#### Transgenic Mice

##### Preparation of Transgene Constructs and Generation of Transgenic Mice

Codon-optimized human RS1 cDNA coupled to mini-mGluR6 promoter was synthesized and microinjected into pronuclei of one cell stage fertilized eggs from the B6.SJL (F1 hybrid) mouse strain. Injected embryos were implanted into pseudo-pregnant CD1 females. F0 live births were under the care of the same foster moms until weaning. Genotyping was conducted by PCR amplification of the human RS1 cDNA in the transgene. Screening primers are selected from the hRS1 National Center for Biotechnology Information reference sequence: NM_000330.4 (CDS: 41-715): (1) hRS1-F (nt 62-84): TTTTGTTATTACTTCTCTTTGGC; and (2) hRS1-R(nt 650-632): GCGGGATGAGGCGGATGAA. Positive F0 founders were mated to wild type C57Bl6/J from The Jackson Laboratories for several generations until they reached congenic state with C57BL/6J. Hemizygous males of the transgenic strain were mated to homozygous *Rs1*-KO females to generate a colony of transgenic mice encoding human RS1 gene under the control of the mouse mini mGluR6 promoter (mGluR6*-RS1+/+ Rs1^−^/y).

### Virus Injections

Either subretinal or intravitreal injections were performed in eyes of XLRS (Rs1-KO) mice at 14 days of age. XLRS mice were anesthetized by intraperitoneal injection of ketamine (100 mg/kg) and xylazine (10 mg/kg), and one drop of 0.5% tetracaine topical anesthetic was applied to the cornea. For subretinal injections, the mouse was placed under a dissecting microscope and the cornea was positioned upward. The conjunctiva membrane was removed with scissors and forceps. A 33G sharp needle was used to incise the sclera down to the subretinal space 1mm from the temporal limbus, and a blunt 35G needle was inserted through this hole to inject 1.0 μL of rAAV8-Ple155-RS1 vector suspension in saline at a concentration of approximately 2 × 10^12^  vg/mL. For intravitreal injections, 1.0 µL of rAAV2/2-Y444 – mini-mGluR6-RS1 vector suspension in saline at a dose of 2.19 × 10^13^ vg/mL or 0.5 µL rAAV2/2-Y444 – mini-mGluR6-mCherry vector suspension in saline at a dose of 2.09 × 10^13^ vg/mL was injected into the vitreous body through the sclera on the nasal side of the eye approximately 1 mm posteriorly to the limbus with a 10-μL NanoFil syringe tipped with a removable 35G needle (World Precision Instruments, Inc., Sarasota, FL, USA). Triple antibiotic ophthalmic ointment was applied to the injection site. In both cases, the contralateral eye remained untouched and served as a control. All parameters of retinal structure and function were evaluated approximately 5 weeks after viral injection.

### Optical Coherence Tomography (OCT)

Schisis cavities were observed in retinal cross-sectional images using OCT imaging system (Envisu R2200-SD-OCT; Bioptigen, Durham, NC, USA) under anaesthesia.^41^ The pupils were dilated with a mixture of tropicamide and phenylephrine. Artificial tears (Alcon Laboratories, Inc., Fort Worth, TX, USA) were applied to maintain corneal hydration and clarity. Two horizontal linear B-scans (1000 A-scans per B-scan), each the average of 10 frames, were obtained from nasal to temporal poles through the optic nerve head. Radial volume scans consisting of 10 B-scans were collected at 18° intervals. Rectangular volume scans of retinal thickness across approximately one-third of the central retina were collected with 100 B-scans.

### Electroretinography

Full-field ERG responses were recorded (Espion E2 system with ColorDome Ganzfeld; Diagnosys LLC, Lowell, MA, USA). Animals were dark adapted overnight for 12 hours and then anesthetized under dim red light with intraperitoneal ketamine (80 mg/kg) and xylazine (10 mg/kg). Pupils were dilated with topical 0.5% tropicamide and 0.5% phenylephrine HCl. The cornea was kept moist throughout with methylcellulose solution. The gold recording wire electrode was placed on the cornea, and a gold reference electrode was placed in the mouth. The circuit ground electrode was a subcutaneous needle placed close to the tail. ERGs were recorded simultaneously from both eyes at bandwidth of 0.1 to 500 Hz. Dark-adapted ERGs were evoked by white flashes (–5.8 to 0.7 log scotopic candela-sec/meter2 [sc cd-s/m^2^]), with interflash intervals of 3 seconds for the dimmest intensities, up to 60 seconds for bright flashes (0.7 log sc cd-s/m^2^). Light-adapted photopic ERGs were evoked with flashes (0.0 to 2.0 log sc cd-s/m^2^) presented on a steady background of 34 sc cd/m^2^, after an initial 5-minute exposure to this background. A-wave amplitudes were measured from baseline to trough, and the b-wave was measured from a-wave trough (when present) to b-wave peak, after subtracting oscillatory potentials isolated with a digital band-pass filter (45–500 Hz). ERG amplitudes in response to a stimulus intensity of –0.82 log sc cd-s/m^2^ were used to compare treated and untreated eyes in dark-adapted conditions; at this flash intensity, the ERG responses are mainly rod driven. The effect of treatment on cone mediated responses were assessed at a stimulus intensity of 1.0 log sc-cd·s/m^2^ in light-adapted conditions.

### Immunohistochemistry

Mouse retinas were dissected, fixed, and cryosectioned at 10 μm thickness by standard protocols as described earlier for immunohistochemistry.^21^ The following antibodies were used: rabbit polyclonal antibody against the N-terminus of RS1 (amino acid residues 24–37 [1:1000; Custom made, ThermoFisher Scientific, Waltham, MA, USA]^33^; monoclonal antibody against the protein kinase C-alpha [1:500, Santa Cruz Biotechnology, Dallas, TX, USA]; mouse monoclonal antibody against glutamine synthetase [1:1000; Sigma-Aldrich, St. Louis, MO]; calretinin [1:2000; BD Biosciences, San Jose, CA, USA]; goat anti-mCherry [1:2000; Biorbyt, Cambridge, UK], and 1:1000 mouse monoclonal Na+/K+ ATPase [alpha-3 subunit] antibody [Na/K-ATPase alpha 3; ThermoFisher Scientific], secondary antibody conjugated to Alexa Fluor 568 dye or Alexa Fluor 488 dye [ThermoFisher Scientific]). Retinal images were obtained and processed with a Nikon C2 confocal microscope with Advanced Element software (Nikon, Tokyo, Japan). Image analysis was performed using image-editing software (Photoshop CS6; Adobe Systems, Inc., San Jose, CA, USA).

## Results

### Mini-mGluR6 Promoter (In4s-In3-200En-mGluR500P)

**Figure 2.**
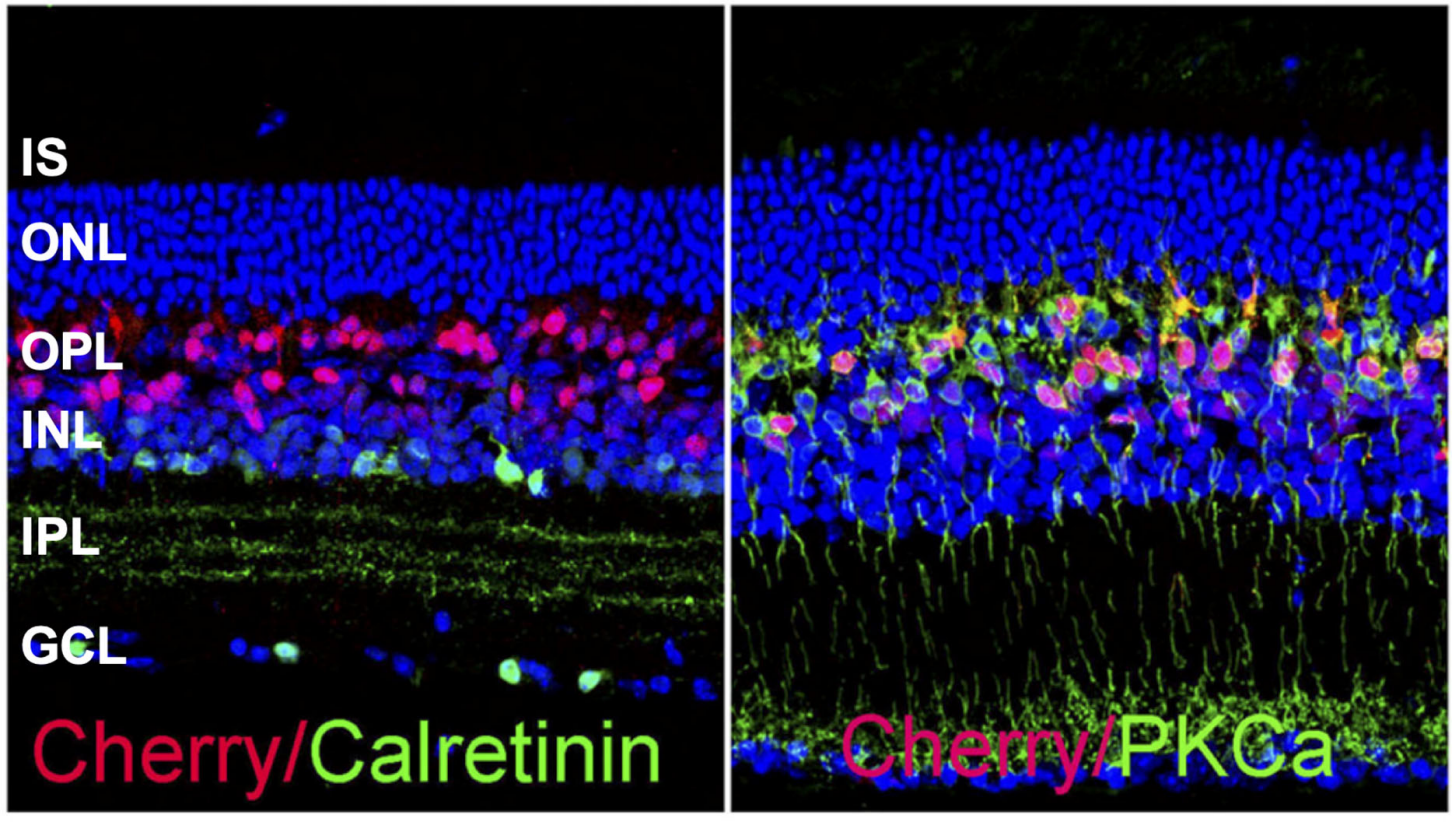
**In XLRS mouse, AAV2/2-Y444F–mediated mCherry expression is restricted to cells in the inner retina**. Retinal expression of fluorescent transgenes mCherry was examined 5 weeks after intravitreal injection of In4s-In3-200En-mGluR500P-mCherry (mini-mGluR6-mCherry) into XLRS mouse retina. mCherry expression was restricted to cells in the inner retina, most predominately in RBCs. There is no labeling of mCherry in the photoreceptor layer. Vertical sections of XLRS mouse retinas were colabelled for mCherry (*red*), protein kinase Cα (*green*), RBC marker; calretinin (*green*), amacrine and ganglion cell marker. DAPI was used as a nuclear counterstain (*blue*).

We first confirmed the specificity of the promoter for bipolar cell targeting by evaluating expression of red fluorescent protein mCherry driven by the mini-mGlur6 promoter. Intravitreal injections of rAAV2/2-Y444F virus vector encoding the fluorescent reporter construct (mini-mGluR6-mCherry) into XLRS mouse retina did not show any expression of mCherry either in photoreceptor soma or in photoreceptor inner and outer segments (Fig. 2). Double immunostaining with antibodies against protein kinase Cα, a marker of rod bipolar cells (RBCs), showed that mCherry-positive cells (targeted by the vector) were predominantly RBCs. An earlier study had shown that this promoter can also target cone ON bipolar cells in marmoset but not significantly in mice.^25^ Double immunostaining with antibodies against calretinin showed no off-target labeling of ganglion cells or amacrine cells (Fig. 2). These results confirm that the mini-mGluR6 promoter enables gene expression in XLRS mouse retina mainly restricted to RBCs of the inner retina.

### rAAV-Mediated RS1 Expression Under Mini-mGluR6 Promoter

**Figure 3.**
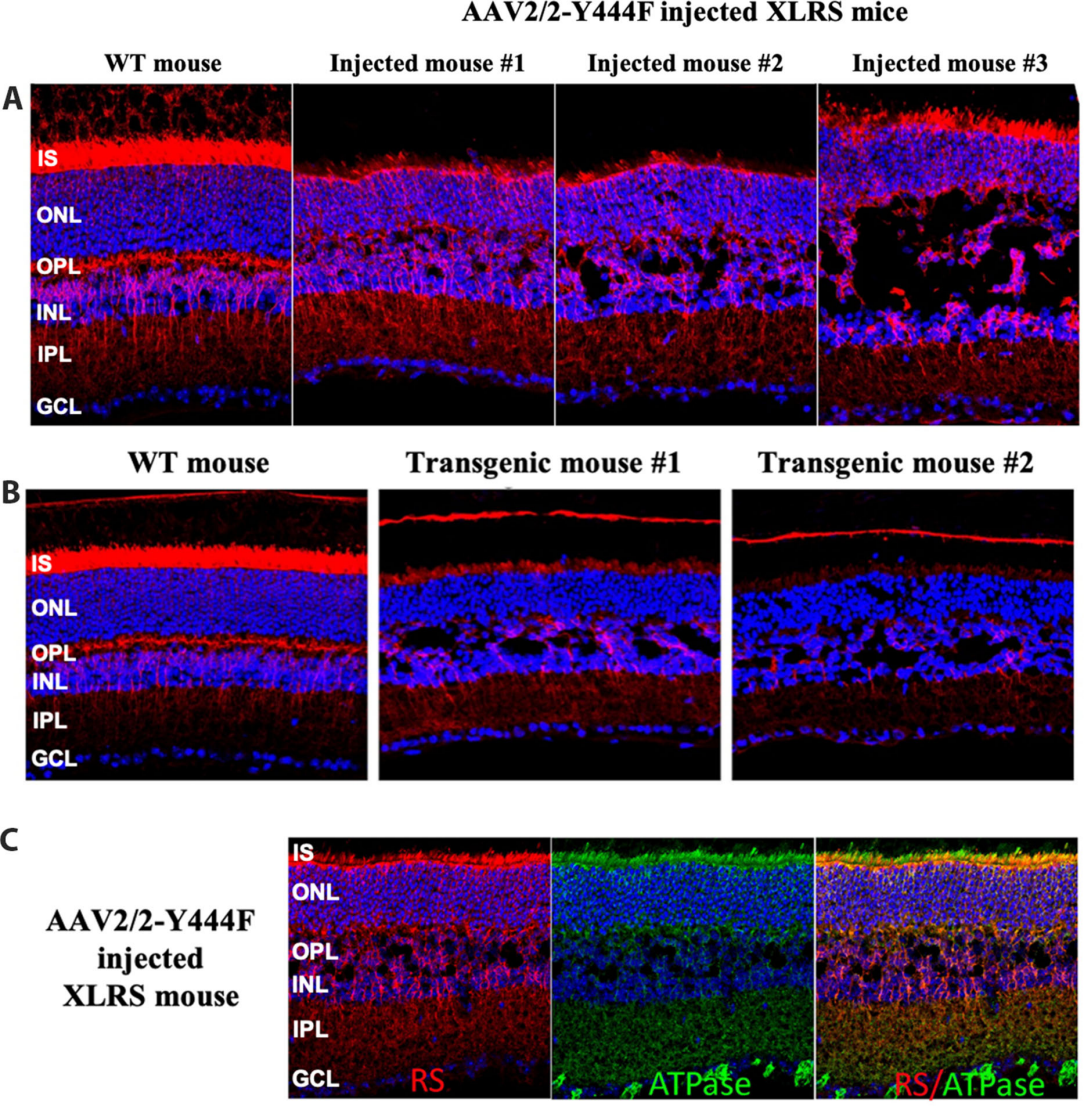
(**A**) **RS1 expression variability and schisis cavity closure in XLRS mouse retina under mini-mGluR6 promoter**. RS1 expression in WT and XLRS mouse retinas 5 weeks after intravitreal injection of AAV2/2-Y444F vector encoding human RS1 gene under bipolar cell specific mini-mGluR6 (In4s-In3-200En-mGluR500P) promoter. Retinal sections from injected XLRS mice along with age matched C57BL/6 WT mice were immunolabeled with antibodies raised against RS1 (*red*), Na/K ATPase a3 subunit (*green*), and DAPI counterstain for nuclei (*blue*). In WT retina, Rs1 is profusely present in the photoreceptor ISs, outer plexiform layer (OPL), bipolar cell layer, and IPL. In XLRS mouse retinas, intravitreal injection of viral vector leads to RS1expression specifically in inner retina with Rs1 immunoreactivity in cell processes within the IPL with moderate to modest cavity closure. RS1 localization is also seen in IS of photoreceptors. Retina 3 has the least cavity closure even though it has the most RS1 labeling. (**B)** RS1 expression in transgenic mice under mini mGluR6 promoter. In transgenic mice the mini-mGluR6 promoter drives RS1 gene expression in the inner retina but the transgenic mice tended to have significantly more variability in gene expression levels with no effect on cavity closure. RS1 labeling is also seen in photoreceptor IS. Creation of transgenic mice is described in Materials and Methods section. (**C)** RS1 localization on photoreceptor IS. Mini-mGluR6-RS1 injected XLRS mice retinas showed immunolabeling of Na/K ATPase (*green*) in photoreceptor IS, OPL and IPL, the labeling being intense in IPL. Merged images confirmed a high degree of colocalization of the Na/K ATPase (*green*) and RS1 (*red*) in all layers of the retina.

Intravitreal injection of rAAV2/2-Y444F virus vector with the mini-mGluR6-RS1 construct resulted in RS1 expression throughout the whole retina as previously reported.^25^ RS1 expression was seen in cells beyond just RBCs (Fig. 3A), including photoreceptor inner segments (IS), radial-oriented structures in the ONL, and in the outer plexiform layer. The cavity severity showed considerable heterogeneity across the injected XLRS retinas.

This mini-mGluR6 promoter was also used to create three transgenic mouse lines expressing *RS1* gene under the control of the mini-mGluR6 promoter. RS1 immunohistochemistry with these transgenic mice showed RS1 staining predominantly in the inner retina and photoreceptor IS with no effect on cavity closure. There was considerable variability in transgene (*RS1*) expression levels across the three transgenic mouse lines generated using the same construct (Fig. 3B).

### RS1 Is Localized in Photoreceptor IS

The primary objective of this study was to restrict RS1 expression to the inner retina and thereby decouple RS1 effects expressed in bipolar cells from RS1 in photoreceptors. Finding RS1 on photoreceptor IS (Figs. 3A, B) was surprising because the mini-mGluR6-RS1 promoter usually gave expression restricted to RBCs of inner retina (Fig. 2), and this was thought likely from diffusion of RS1 from the inner to the outer retina. The diffusing pool of RS1 seems to concentrate on the cell surface. Previous studies proposed that the Na/K ATPase (α3β2) isoform anchors RS1 to the surface membranes of both photoreceptors and bipolar cell and that it is involved in RS1 functional interactions.^42^^,^^43^ Consistent with this concept, RS1 was colocalized with the α3 subunit of Na/K ATPase on photoreceptor IS membrane surfaces (Fig. 3C), but because localization to a photoreceptor IS was not a desirable feature for this study, we excluded the mini-mGluR6-RS1 injected mice and mini-mGluR6 -RS1transgenic mice from analysis.

### AAV8-Ple155-RS1

**Figure 4.**
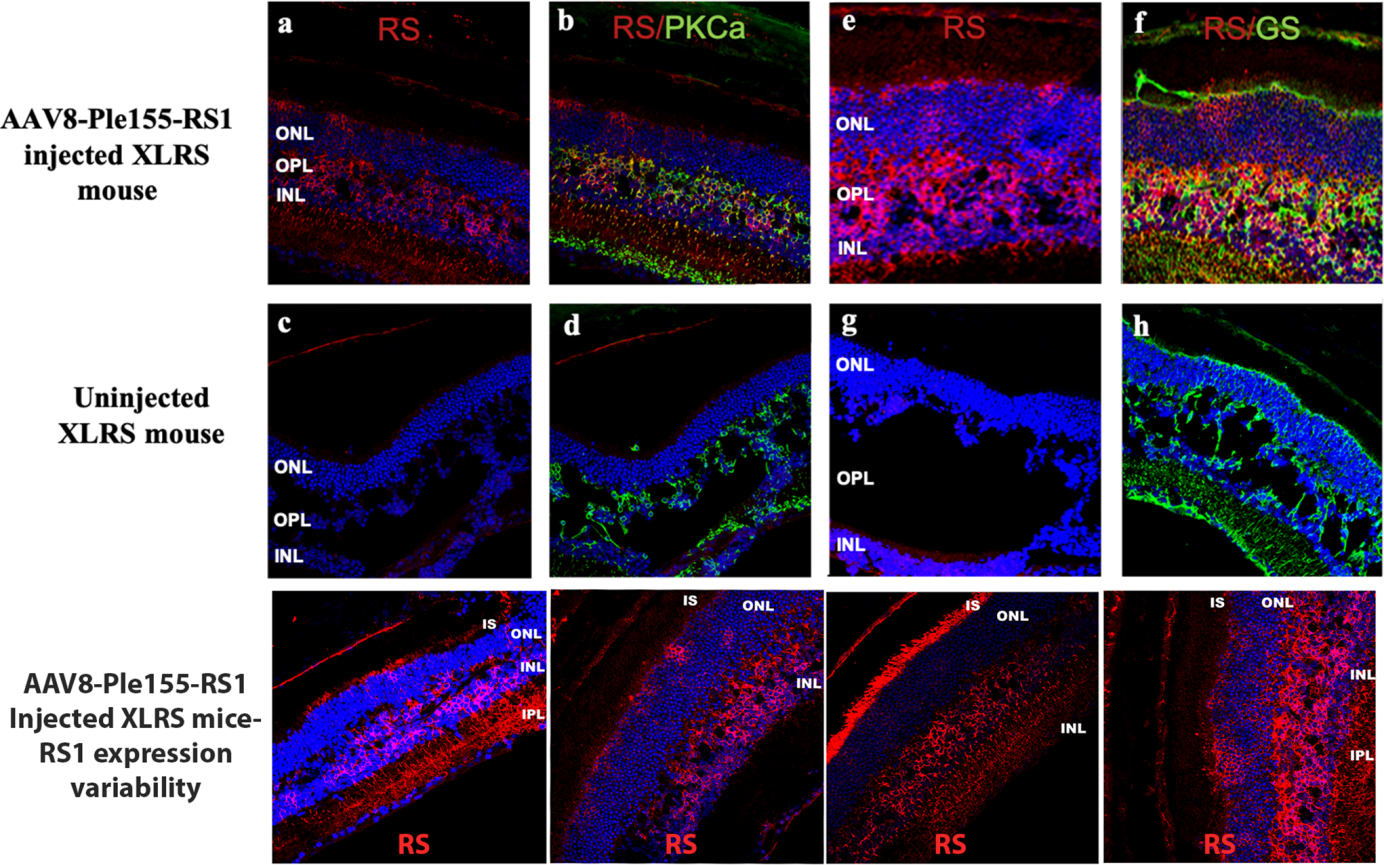
**MiniPromoter Ple155 drives bipolar cell specific RS1 gene expression in XLRS mice retinas**. Retinal sections from representative vector injected and uninjected XLRS mice 5 weeks after subretinal injection of AAV8-Ple155-RS1 vector were immunolabeled with anti-RS1 antibody (*red*), RBC marker protein kinase Cα (*green*), Müller glia marker glutamine synthetase (GS), (*green*) and DAPI counterstain for nuclei (*blue*). Subretinal injection of AAV8-Ple155-RS1 at postnatal day 14, led to robust RS1 expression in bipolar cells (**a**, **e**) as confirmed by costaining with the bipolar cell marker PKCα (**b**). Compared with uninjected mice retina (**c**, **d**), which showed no RS1 labeling and displayed large schisis cavities, injected retinas showed schisis closure and marked improvement in inner retina structure. In AAV 8-Ple155-RS1 injected retinas, RS1 staining juxtaposed in close physical association with parallel Müller cell processes as revealed with anti-GS staining (**f**, **h**). Representative images for 4 mice in each group. The images in the lower panel show RS1 expression variability and photoreceptor localization in Ple155 injected retinas (*n* = 4).

Next, we evaluated the MiniPromoter Ple155 construct driving RS1 expression in XLRS mouse retina bipolar cells by subretinal injection of rAAV8-Ple155-RS1. This construct gave substantial RS1 expression in the inner retina (Figs. 4a, e), and RS1 and protein kinase Cα antibodies showed colocalized immunolabeling, indicating that these are RBCs (Fig. 4b). However, the results were not perfect; 2 of the 10 injected retinas showed some RS1 at photoreceptor IS (Fig. 4, lower panel). To test our hypothesis regarding the sufficiency of bipolar expression to decrease schisis, we selected those retinal sections or regions that showed minimal or no RS1 on photoreceptor IS. The uninjected eyes had large retinoschisis cavities across the entire retina (Figs. 4c–h), whereas injecting the of rAAV8-Ple155-RS1vector gave areas of substantial RS1 expression by RBCs and showed conspicuous improvement in the inner retinal layer structure (Figs. 4a–f).

Müller cell bodies in the INL have a close relationship with neurons and the major blood vessels and form architectural support structures. We assessed the morphology of Müller cell arborization in retinal cryosections.^44^^,^^45^ In injected retinas, RS1 staining was juxtaposed closely with parallel Müller cell processes identified by glutamine synthetase staining (Fig. 4f). Müller cell are densely packed toward the center of the retina for anatomical support to retinal neurons. Imaging the Müller cell staining showed structural recovery of the inner retina, and the integration and alignment of RS1-expressing bipolar cells with the neighboring cells.

Some previous studies noted that each rAAV vector has a unique pattern of transduction and cell type–specific expression. Lu et al.^25^ reported that viral vectors with rAAV2/2-Y444F and rAAV2/7m8-Y444F capsids predominately target mouse RBCs, whereas in marmoset retina they target RBCs and cone ON bipolar cells. For Ple155, the Boye Laboratory reported that nyctalopin expression in ON BCs was widespread throughout the mouse retina after intravitreal injection with rAAV2-based vectors but not with rAAV8(Y733F) vectors.^27^ Because we did not examine cell type–specific differences within the inner retina, it is difficult to determine extent of ON bipolar cell transduction in our experimental system.

### RS1 Expression Variability and Effect on Cavity Closure

**Figure 5.**
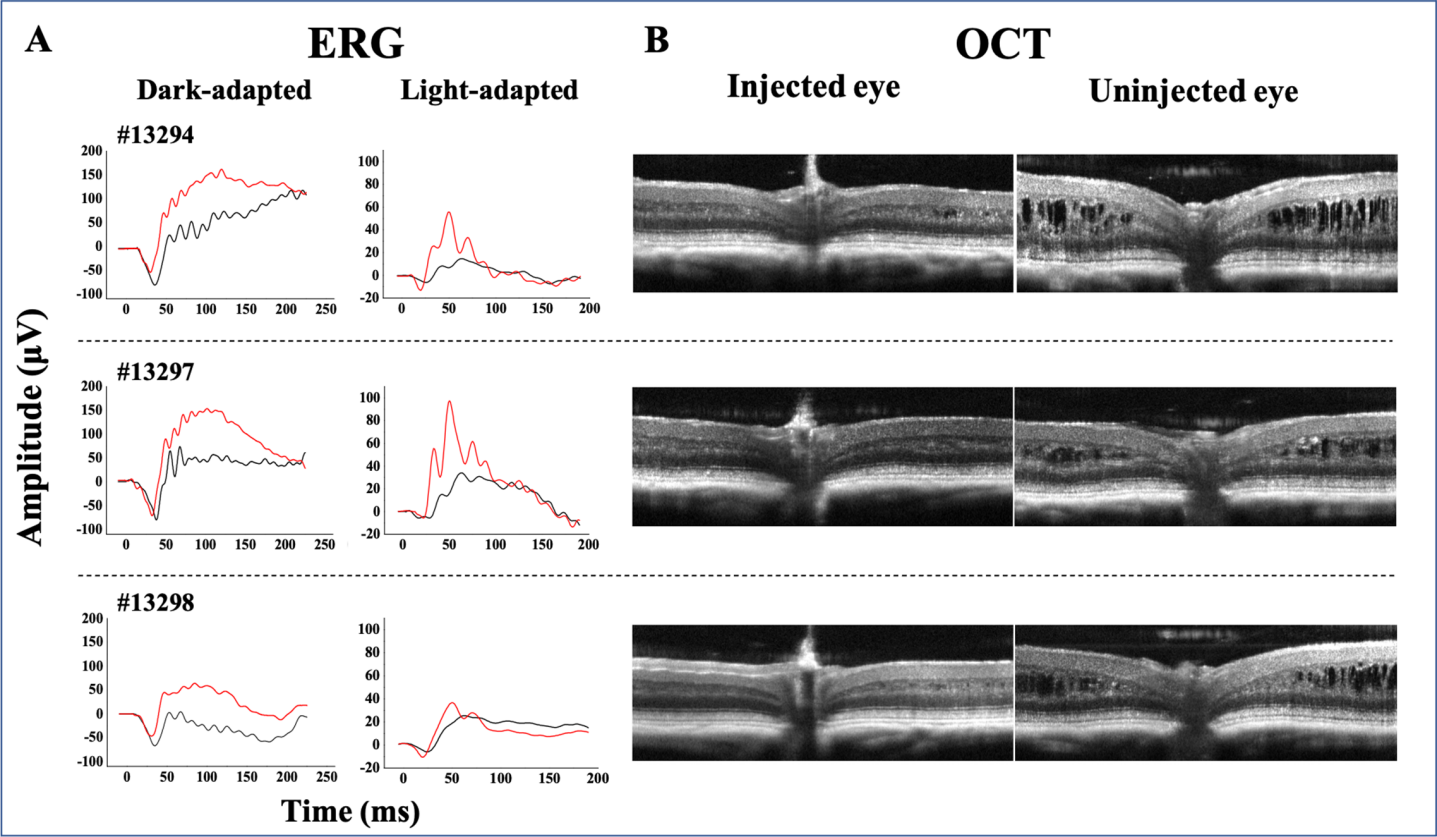
**Functional and structural changes in XLRS mice retinas after injection of AAV8 vector encoding RS1 under Ple155 promoter**. (**A**) Representative ERG waveforms recorded in scotopic and photopic conditions in 3 XLRS mice between 5 and 6 weeks after unilateral subretinal injection of AAV8-PLe155-RS1. The flash luminances at which the dark- and light-adapted ERGs shown in the figure were elicited were −0.82 log sc cd-s/m^2^ and 1.0 log sc cd-s/m^2^, respectively. Both scotopic and photopic ERG responses in the injected eye (*red waveforms*) were increased in amplitude when compared with the uninjected eye (*black waveforms*). All three study animals showed larger scotopic and photopic b-wave amplitudes and a shorter scotopic a-wave implicit time in the treated eyes, indicating a selective improvement of the postphotoreceptorial element function, including the photoreceptor–bipolar cell synapse. The a-wave amplitude is measured from baseline (0 ms) to the negative trough; b-wave amplitude is measured from the a-wave trough to the positive peak. Stimulus flash occurs at 0 ms. (**B**) Analysis of retinal structure by OCT in the same 3 animals showed a reduction in intraretinal cystic cavities in the eye injected with the vector and almost complete restoration of the normal retinal architecture.

The effects of RS1 treatment on inner and outer retinal morphology was evaluated in 10 treated XLRS mice by high-resolution spectral domain OCT imaging of retinal cavities (Supplementary Fig. S3). Untreated eyes showed INL schisis with cystoid lesions and laminar dehiscence between the INL and outer plexiform layer. The AAV8-Ple155-RS1 vector gave variable results in part due to limitations of injecting uniformly into the small mouse eyes and from viral transduction efficiencies. Further, even the uninjected XLRS eyes show variation of retinal schisis cavities. This makes quantitative evaluation tenuous, but several qualitative statements can be made:

• In a side-by-side comparison of injected versus uninjected eyes of the 10 XLRS mice, all injected eyes had similar or fewer cavities and better lamellar structure compared with the respective uninjected contralateral eye.
• Only one untreated eye (#13301) showed virtual absence of cavities, and this was true also for the contralateral treated fellow.
• Five injected eyes achieved minimal or no cavities (#13294, 13297, 13298, 13299, 13301), whereas only 2 of 10 uninjected eyes showed schisis cavities of minimal size and extent (#13301, 13302).
• Three injected eyes achieved high levels of bipolar RS1 expression (#13294, 13297, 13298), and these had substantially better retinal structure than the contralateral eyes, with the outer plexiform layer and INL margins were more distinct (Fig. 5B).
• These qualitative analyses support the hypothesis that expression of RS1 by bipolar cells is sufficient to decrease or close cavities.

### AAV8-Ple155-RS1 Vector Ameliorates Inner Retinal Dysfunction and Structure

ERG responses of the three XLRS mice, with robust inner retinal RS1 expression by the AAV8-Ple155-RS1 vector (Fig. 5A) showed improvement of inner retina function, indicated by increased amplitudes of both dark- and light-adapted b-wave responses, and the increased b-/a-wave ratio in the treated eye. The dark- and light-adapted b-wave amplitudes on average increased by 47% and 121% in injected eyes compared with the uninjected eyes (mean, 190 µV [range, 131–227 µV] vs mean, 129 µV [range, 50–188 µV] and mean, 66 µV [range, 48–93 µV] vs mean, 30 µV [range, 20–39 µV], respectively). Dark-adapted a-wave implicit time^46^ was on average 4.87 ms shorter in the injected eyes compared with the fellow eyes (mean, 30.87 [range, 30–32.2] vs mean, 35.73 [range, 34.6–37.6]), indicating improved synaptic transmission between photoreceptors and bipolar cells. The b-/a-wave ratio (which normalizes the b-wave by the photoreceptor response and estimates the effect on the isolated postsynaptic responses) was consistently about twice as large for injected eyes versus uninjected eyes (mean, 3.39 [range, 2.85–4.24] vs mean, 1.71, [range, 0.75–2.38]). As expected, the dark-adapted a-wave amplitudes did not change after vector injection at flash intensities (mean, 57 µV [range, 46–74] vs mean, 74 µV [range, 67–79]), suggesting no detectable effect of RS1 on photoreceptor function.

## Discussion

The study indicated that RS1 targeted to bipolar cells in the INL with the Ple155 promoter was able to improve the schisis spaces leading to recovery of inner retinal structure and synaptic function independent of photoreceptor RS1 expression.

### RS1-Bipolar Neurons

Bipolar cells are the first retinal interneurons that relay signals of the outer retinal, sensory photoreceptor neurons to RGCs and amacrine cells in the proximal retina. West and Cepko identified 15 subtypes of bipolar interneurons (rod bipolar and several OFF cone and ON cone bipolar subtypes) in the mouse retina, each with characteristic gene expression, morphology, and light responses.^47^^–^^49^ Initial work with RS1 considered that it was preferentially taken up from photoreceptors and carried into the inner retina by Muller cells,^50^ but this observation has not been proven in vivo. The Molday laboratory demonstrated that Rs1 is synthesized locally in bipolar cells.^13^ Immunofluorescence analysis of 2-month-old and 1-year-old rd/rd mouse retinas, which lack rods and most cones showed Rs1 on bipolar cells, comparable with WT retina. Loss of structural integrity of bipolar cell layer and disruption of bipolar cell synaptic processes are early events in XLRS pathology.

In the present study, an immunohistochemical analysis of RS1expression showed that both mini-mGluR6 and Ple155 promoters gave bipolar cell specific expression patterns, but to exclude possible off-target effects, retinas with RS1 localization on photoreceptor IS (minimGluR6 promoter) were excluded from the analysis. Even despite variations of vector penetrance, gene transfer efficiency and phenotype variation across injected mice, those retinal areas of robust RS1 expression showed tightly packed bipolar cells, had fewer cavities and showed improved lamination of photoreceptor cells with bipolar cells. The retinal OCT sections showed a modest decrease of ONL thickness that, without treatment, would have progressively diminished by natural history. Closure of large schisis cavities is a first step toward restoring synaptic integrity between photoreceptors and bipolar cells^21^ and for neuroglial circuit formation.^51^

Earlier studies including our own showed that targeted photoreceptors using cell specific promoters, for example, RS1, or opsin, or 7m8-rho, gave robust RS1 expression and intense immunofluorescence labeling of photoreceptor IS.^21^^,^^52^^,^^53^ These photoreceptor specific promoters also resulted in greater RS1 localization on bipolar cells than the current bipolar cell specific promoters, as RS1 seems to diffuse readily from photoreceptors to the bipolar cell layer. However, RS1 staining intensities in both photoreceptor and bipolar cells were one-half or less than in WT retinas. Additionally, in comparing photoreceptor versus bipolar cell specific promoters, one notes that photoreceptor cells outnumber bipolar cells by at least 10-fold. Like what was observed in this study, the peripheral regions of the retina, distant from the site of injection, exhibited a relative absence of RS1 labeling. Within the length, regions of strong and weak staining grades were observed, and few cavities were still visible. Considering the limitations of the viral transduction and the promoters used in this study, we limited our analysis to bipolar cells in areas of robust RS1 expression that showed no or minimal photoreceptor localization.

### RS1 Diffusion

The bipolar cell–specific RS1 expression is tightly regulated by transcriptionally targeted cell-specific promoters (mini-mGluR6 and Ple115), and RS1 localizing at nontargeted photoreceptors results from RS1 diffusion from the inner retina. Conversely, as defined elsewhere in this article, RS1 diffused from the outer retina into the inner retina, even when transgene expression was restricted to photoreceptors.^52^^,^^53^ It is likely that overexpressing a secreted protein such as the RS1 results in diffusion away from the high densities regions to reach its normal extracellular target sites. From the present work, it seems that RS1 diffusion is bidirectional between bipolar cells and photoreceptors, at least in degenerated XLRS mouse retina. In intact retina, under normal conditions, the high affinity of RS1 for photoreceptor and bipolar cell membrane surfaces would restrict its diffusion into the extracellular matrix and may be the reason that, despite the proximity of the photoreceptor outer and IS, we found no evidence by electron microscopy that RS1 is present on connecting cilium and outer segment membranes.^15^ The Flannery Laboratory explored whether diffusion of RS1 expressed by transduced Müller cells could offer a treatment strategy, but Müller cell expression failed to achieve long-term rescue, as that occurred only when expressed by photoreceptors or with added expression by other inner retinal neurons.^53^

### Photoreceptor Heterogeneity in XLRS Disease

If bipolar cell expression of RS1 can rescue from schisis, what would be the function of RS1 in photoreceptors? Cell packing in the INL and ONL are different and "schisis" in the photoreceptor layer may not occur because the interphotoreceptor matrix fills this highly compartmentalized extracellular space. Studies of XLRS mouse models showed that schisis-related inflammation leads to development of photoreceptor degeneration.^6^^,^^24^^,^^54^^,^^55^ Inflammatory stimuli promote photoreceptor degeneration by their cytotoxic effects,^56^ and a recent exploratory study indicated that XLRS subjects exhibit a baseline proinflammatory immune cell phenotype.^4^ The early age loss and dysfunction of photoreceptors occurs in both mouse models and some patients with XLRS. As a majority of patients with XLRS maintain normal ERG a-wave amplitude, and hence intact phototransduction, even with morphological changes, the question remains of the role of RS1 for proper photoreceptor function.^57^

### Molecular Basis for XLRS Photoreceptor Pathology

Investigations have focused heavily on the interaction of RS1 with the retina specific isoform of Na/K-ATPase, a plasma membrane enzyme.^42^^,^^43^^,^^58^^,^^59^ The Molday Laboratory proposed that the binding of RS1 to β2 subunit (ATP1B2) of retinal Na/K-ATPase may directly regulate sodium pump activity in maintaining fluid balance within photoreceptor and bipolar cell layers,^10^ and loss of functional RS1 could lead to fluid accumulation in the extracellular environment in the form of fluid-filled cystic retinal schisis cavities observed in patients with XLRS and in mouse models. The voltage-gated potassium (Kv) channel subunits Kv2.1 and Kv8.2 were also identified as interaction partners of the retinal Na/K-ATPase (Schmid et al., 2021ARVO-E-Abstract, IOVS;62:3053). However, assessing the retinal Na/K-ATPase ion pump activity, by measuring rubidium cation (Rb^+^) transported into cells,^43^ or by patch clamp analysis of Kv2.1– and Kv8.2–mediated potassium ion currents failed to identify a direct effect of RS1 on Na/K-ATPase–mediated ATP hydrolysis on Kv channel-mediated potassium ion currents.^60^

The Friedrich Laboratory subsequently proposed that RS1 regulates Na/K-ATPase localization and intracellular signaling, and that. In XLRS, defective compartmentalization of the retinal Na/K-ATPase and its complexing partners, namely, the Kv ion channels, represents an initial step in dysregulated fluid homeostasis between the intracellular and extracellular environments of the photoreceptor and bipolar cell layers.^43^^,^^60^ Electron microscopy also showed IS membrane changes in XLRS mouse retina.^15^^,^^41^ Whereas photoreceptor IS and OS were closely packed and well-organized in the WT retina, IS membranes in the XLRS mouse retina were irregular, with large gaps between adjacent cells. The shape and structure of XLRS photoreceptor mitochondria were also markedly altered. IS membrane destabilization in the absence of functional RS1 disrupts the spatial and functional coupling of membrane channels and signaling proteins leading to loss of retinal cell homeostasis.

Our results in the XLRS mouse show that expressing RS1 only in bipolar cells can rescue spatial relationship between bipolar cells and ameliorate schisis pathology independent of photoreceptor expression. The prediction that RS1 is an adhesion molecule was built based on the property of the discoidin domain and corroborative evidence of retinal layer splitting seen in patients with XLRS and mouse models, by implication or inference. In cellular slime mold D. discoideum, discoidin I accumulates during aggregation concurrent with increased cell-cell adhesiveness (Fig. 1E). RS1 is expressed postnatally when photoreceptors and bipolar cells are born and neurons are organizing into retinal layers. Cell adhesion plays a crucial role in establishing and maintaining the structural integrity of the retina. However, we still do not know whether the schisis cavities develop exclusively from adhesion failure alone, or whether other mechanisms may contribute, such as abnormal water redistribution due to failure of Müller cell channels, and that providing RS1 protein repairs this defect. We expect that application of new advances in areas probing cell–ECM interactions would provide molecular insight into RS1–extracellular matrix mechanics.

## Supplementary Material

Supplement 1
