# Supplementary material for: Targeted Expression of Retinoschisin by Retinal Bipolar Cells in XLRS Promotes Resolution of Retinoschisis Cysts Sans RS1 From Photoreceptors
Source: Invest Ophthalmol Vis Sci. 2022 Oct 13;63(11):8. doi: 10.1167/iovs.63.11.8 (PMC9583743; doi:10.1167/iovs.63.11.8)
Supplement: Supplement 1 [file iovs-63-11-8_s001.pdf]

SUPPLEMENT: IOVS-22-35014R2

Targeted expression of retinoschisin by XLRS mouse retina bipolar cells promotes the resolution of retinoschisis cysts

Camasamudram Vijayasarathy, Yong Zeng, Dario Marangoni , Lijin Dong , Zhuo-Hua Pan, Elizabeth M. Simpson, Robert N. Fariss, Paul A. Sieving.

Supplemental Figure 1

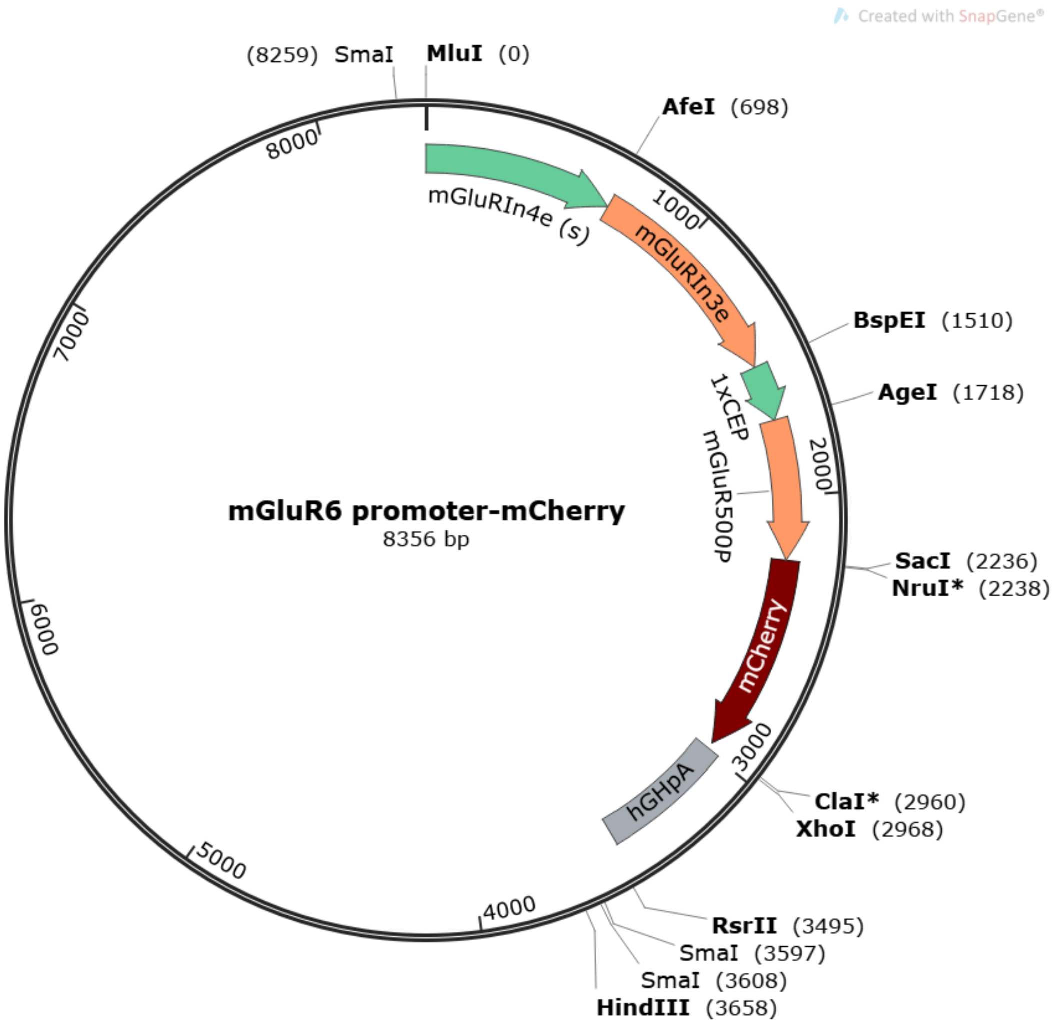

Supplemental Figure 1. Map of mGluR6 promoter-mCherry vector and RS1 cloning

MGluR6-mCherry vector map (Lu et.al., 2016). mCherry cDNA was replaced with human RS1 cDNA. The human RS1 cDNA (NCBI Reference Sequence NM_000330.4, nucleotides 41-715) was synthesized (GeneScript, Piscataway, NJ, USA) with overhangs that reconstitute the SacI and XhoI sites and inserted into expression vector mGluR6-mCherry to replace mCherry by standard cloning protocols.

Supplemental Figure 2

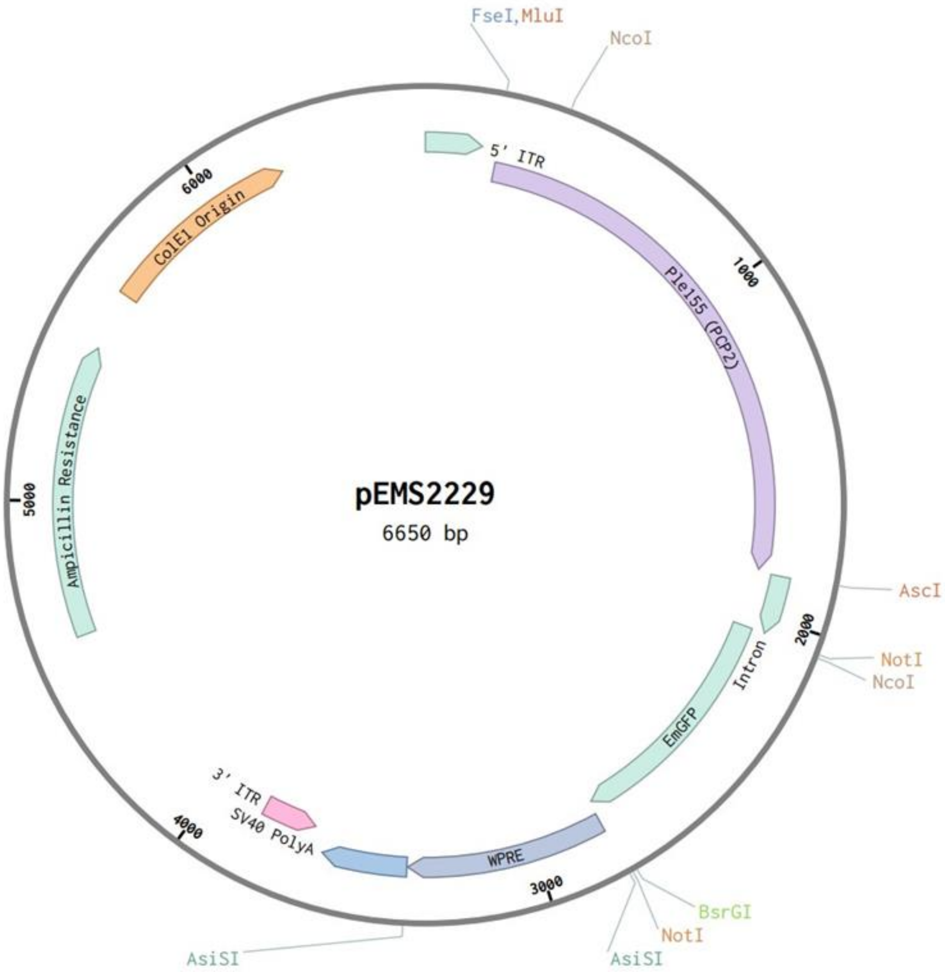

Supplemental Figure 2. Map of pEMS2229 vector and RS1 cloning

pEMS2229 vector map (Plasmid ID:183056, Addgene, Watertown, MA). In the pEMS2229 vector EmGFP was replaced with human RS1 cDNA. The human RS1 cDNA (NCBI Reference Sequence NM_000330.4, nucleotides 41-715) was synthesized (GenScript, Piscataway, NJ, USA) with overhangs that reconstitute the NcoI and BsrGI sites and inserted into expression vector pEMS2229 by standard cloning protocols.

Supplemental Figure 3

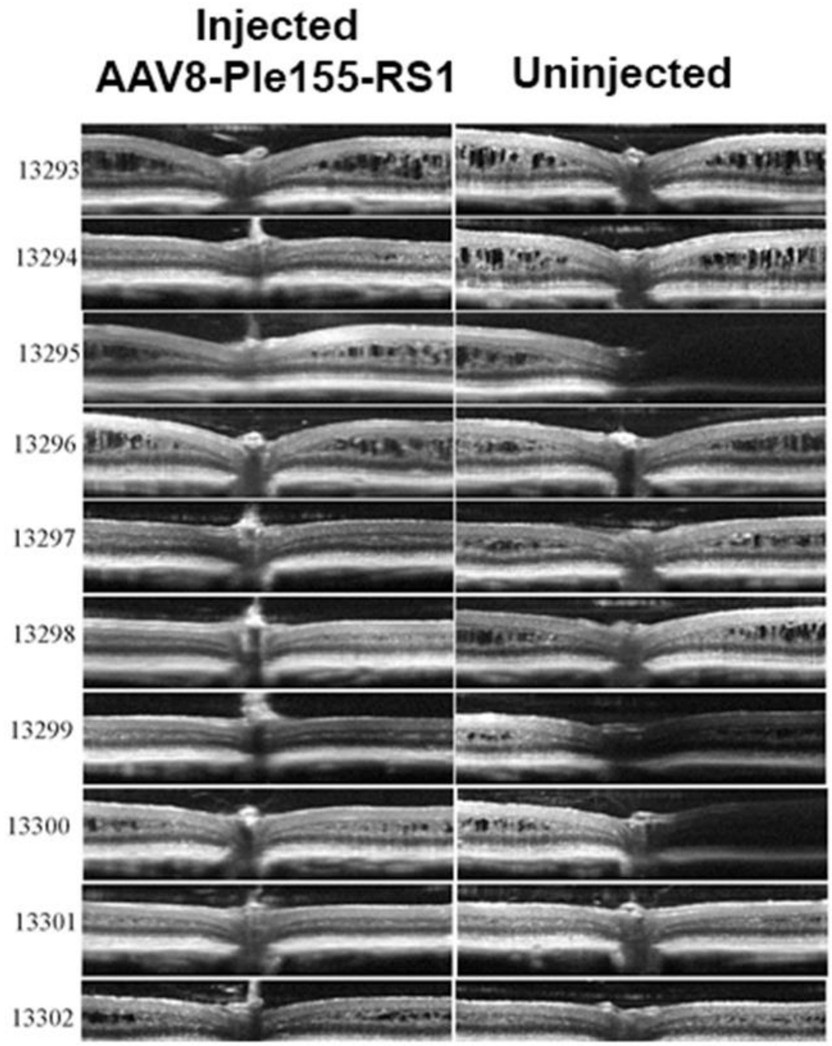

Supplemental Figure 3: SD-OCT scans collected from XLRS mice 35 days after RS1 gene transfer. The scans of both uninjected and injected XLRS mice retinas revealed uneven distribution of cavities and individual variation between mice. However, injected retinas with robust RS1 expression (13294, 97, 98), showed considerably more organized retinal laminar structure, the size of cavities being reduced or completely absent with distinct margin between OPL and INL.
